# Intra-Articular Injections of Polyphenols Protect Articular Cartilage from Inflammation-Induced Degradation: Suggesting a Potential Role in Cartilage Therapeutics

**DOI:** 10.1371/journal.pone.0127165

**Published:** 2015-06-05

**Authors:** Venkatachalam Natarajan, Balaraman Madhan, Moti L. Tiku

**Affiliations:** 1 Council of Scientific and Industrial Research—Central Leather Research Institute, Adyar, Chennai, Tamil Nadu, India; 2 Rutgers, Robert Wood Johnson Medical School, New Brunswick, New Jersey, United States of America; Illinois Institute of Technology, UNITED STATES

## Abstract

Arthritic diseases, such as osteoarthritis and rheumatoid arthritis, inflict an enormous health care burden on society. Osteoarthritis, a degenerative joint disease with high prevalence among older people, and rheumatoid arthritis, an autoimmune inflammatory disease, both lead to irreversible structural and functional damage to articular cartilage. The aim of this study was to investigate the effect of polyphenols such as catechin, quercetin, epigallocatechin gallate, and tannic acid, on crosslinking type II collagen and the roles of these agents in managing *in vivo* articular cartilage degradation. The thermal, enzymatic, and physical stability of bovine articular cartilage explants following polyphenolic treatment were assessed for efficiency. Epigallocatechin gallate and tannic acid-treated explants showed >12 °C increase over native cartilage in thermal stability, thereby confirming cartilage crosslinking. Polyphenol-treated cartilage also showed a significant reduction in the percentage of collagen degradation and the release of glycosaminoglycans against collagenase digestion, indicating the increase physical integrity and resistance of polyphenol crosslinked cartilage to enzymatic digestion. To examine the *in vivo* cartilage protective effects, polyphenols were injected intra-articularly before (prophylactic) and after (therapeutic) the induction of collagen-induced arthritis in rats. The hind paw volume and histomorphological scoring was done for cartilage damage. The intra-articular injection of epigallocatechin gallate and tannic acid did not significantly influence the time of onset or the intensity of joint inflammation. However, histomorphological scoring of the articular cartilage showed a significant reduction in cartilage degradation in prophylactic- and therapeutic-groups, indicating that intra-articular injections of polyphenols bind to articular cartilage and making it resistant to degradation despite ongoing inflammation. These studies establish the value of intra-articular injections of polyphenol in stabilization of cartilage collagen against degradation and indicate the unique beneficial role of injectable polyphenols in protecting the cartilage in arthritic conditions.

## Introduction

Arthritic diseases are characterized by pain, stiffness, and joint inflammation, which eventually lead to articular cartilage (AC) destruction and disability. Osteoarthritis (OA) and rheumatoid arthritis (RA) are the most debilitating forms of arthritis[1]. AC is the highly specialized connective tissue responsible for frictionless movement between the articulating joint surfaces and the transmission of loads with a low frictional coefficient [2]. AC lacks blood vessels and lymphatic supply, has a limited capacity for intrinsic healing and repair, and has structural arrangements that are challenging for repair and restoration [3]. Chondrocytes of AC are embedded in a matrix comprising type II collagen (CII) proteoglycans and water [4]. Water comprises 60–80% of the wet weight of cartilage. Biomechanical properties of collagen and proteoglycan provide tensile and cushioning properties of AC, respectively [5].

The destruction of the AC is associated with reduced synthesis of the matrix components by articular chondrocytes and the enhanced breakdown of the matrix by disintegrin and metalloproteinase with thrombospondin motifs (ADAMTs) and matrix metalloproteinases (MMPs)[6]. The degradation of proteoglycan is an early and reversible process, whereas the breakdown of the collagen network in AC by collagenases results in the irreversible destruction of the fibrillar network[4, 7]. The treatment of arthritis involves different combinations of drugs offered at different stages of the disease to control inflammation and swelling by blocking the prime inflammatory processes [8]. To date, no pharmacological intervention offers protection or treatment from destruction of AC in arthritic conditions [9, 10].

Polyphenols, many of which are well known for their antioxidant and anti-inflammatory activities, are consumed as micronutrients in the human diet, with an average consumption of 1g/day[11, 12]. Polyphenols taken orally are extensively metabolized in the intestinal and hepatic systems, and the metabolites in the plasma differ in their biological activities[12]. Polyphenols are also an integral part of traditional medicines for the treatment of arthritis in many countries[11]. Epigallocatechin gallate (EGCG), quercetin (QUE), and catechin (CAT) are the major polyphenols in preclinical research for the treatment of cancer[13, 14], arthritis[15], diabetes[16, 17], cardiovascular diseases[18], and other inflammatory diseases[16]. Tannic acid (TA) extracted from oak trees also has beneficial biological activities in cancer and diabetes [19–21]. Previous findings relating to the role of polyphenols in arthritis mostly elucidate the mechanisms of inhibiting inflammatory cytokines or MMPs [15, 22–28].

The process of vegetable tanning dates back to ancient times. In the process, the conversion of skin/hide (type I collagen) matrix into leather is done through the crosslinking of plant polyphenols (tannin) with the type I collagen matrix. Polyphenols interact with collagen through hydrophobic association and hydrogen bonding. The multiple hydroxyls functional groups present in the polyphenols will have the ability to have hydrogen bonding with the side functional groups and peptide backbone of collagen triple helices [29, 30]. Thus, crosslinked collagen matrices attain stability against enzymatic degradation [31, 32]. Based on conventional wisdom of vegetable tanning, we hypothesize the binding of polyphenols with type II collagen (CII) in AC and prevention of cartilage degradation.

In this study, we demonstrate the binding of polyphenols (EGCG, QUE, CAT, and TA) to collagen in bovine AC explants, leading to stability against collagenases. The bioavailability of polyphenols (including those in the synovial space) through the oral route is probably nonexistent. Therefore, we attempted to study the effect of intra-articular injections of plant polyphenols on cartilage protection through an *in vivo* model of collagen-induced inflammatory arthritis (CIA).

## Materials and Methods

### Materials

Catechin hydrate (CAT), quercetin dihydrate (QUE), epigallocatechin gallate (EGCG), tannic acid (TA), collagenase (type IA), chondroitin sulfate, hydroxyproline, and complete Freund's adjuvant (CFA) were purchased from Sigma Aldrich, India. Sterile scalpel blades (Surgeon, India) were purchased at a local pharmacy. All other reagents were of analytical grade and purchased from HiMedia Laboratories, India.

### Ethics statement

Animal experiments were carried out in strict accordance with the norms of the Committee for the Purpose of Control and Supervision of Experiments on Animals (CPCSEA) in the institutional animal house. The animals were fed a standard commercial diet with water *ad libitum*. The protocol was approved by our Institutional Animal Ethical Committee (IAEC) of the CSIR—Central Leather Research Institute (IAEC No: 03/02/2011b). All the rats were purchased from the National Centre for Laboratory Animal Sciences (NCLAS), Hyderabad, India.

### Articular cartilage explants

Fresh bovine tibio femoral joints were collected from a slaughterhouse. The cartilage surfaces were visually inspected for the absence of degeneration. Cartilage slices in thicknesses ranging 1.2–2.4 mm were dissected from the surfaces (lateral and medial condyles) using a scalpel. The cartilage slices were punched to obtain samples of uniform size and weight. The samples were washed in cold saline, transferred to sterile cold water, and stored at -40°C until use.

### Collagen, glycosaminoglycan, and water content estimation of AC explants

The cartilage samples were grouped and placed in centrifuge tubes containing PBS. They were then utilized for the estimation of water content, collagen, and glycosaminoglycans (GAG). The water content was determined by the difference between the wet and dry weights of the cartilage. The amount of GAG present in the cartilage explants was determined after papain digestion using dimethylmethylene blue (DMMB) dye [33]. Initially, the cartilage explants were incubated overnight at 65°C in 2 mL of papain digest solution (i.e., papain solution [1%], which was prepared by dissolving 1 g of papain in 100-mL PBE buffer [PBE buffer: 100-mM Na_2_HPO_4_, 10-mM EDTA, 5-mM cysteine, 500-mL deionized water, final pH 6.5) for complete cartilage digestion. The digested sample was then centrifuged at 6,000 rpm to remove any insoluble components, and the supernatant was stored at ˗4°C[33, 34]. Chondroitin sulfate obtained from bovine trachea was used as a standard to estimate the amount of GAG. The total amount of GAG was estimated by adding 200 μL of DMMB solution to the volume of 50 μL of papain-digested supernatant and read on a microplate reader at 525 nm. The collagen content was determined using the method reported by Woessner [35]. By estimating the hydroxyproline content of the hydrolyzed cartilage (treated with 6N HCl at 118°C, for 12 h, in sealed hydrolysis tubes) using dimethylaminobenzaldehyde and chloramine T, a standard curve was generated with standard hydroxyproline. The amount of collagen = amount of hydroxyproline determined × 7.4 (conversion factor).

### Thermal stability of AC explants

Differential scanning calorimeter (DSC) analysis is widely used to determine the physicochemical transformations that occur during thermal degradation [36, 37]. The thermal stability of cartilage depends on the distribution of therapeutic (polyphenol) molecules inside the matrix and their interactions with the collagen fibrils of AC. AC explants were treated with polyphenols (200 μM), at 37°C for 48 h, in a shaking incubator. Stock solutions of polyphenols were prepared in PBS except QUE, which was prepared using DMSO as co-solvent at DMSO:PBS of 1:3. AC explants incubated only with PBS and stored at 4°C were used as controls to prevent autolytic degradation of AC at 37°C. The cartilage samples were washed within 24 h of incubation in PBS. After washing, the cartilage samples (with and without polyphenol treatment) were placed on tissue paper to remove excess surface buffer (the moisture of AC is ~65%). They were then weighed and placed in an aluminum pan for calorimetric analysis using TA Instruments Model DSC Q200/NETZSCH DSC 204 F1. The samples were analyzed in the temperature range 25–150°C at a heating rate of 5°C/min [38].

### Enzymatic stability of AC explants

To study the effect of polyphenol treatment on the enzymatic stability of AC, the explants were treated with polyphenols dissolved in PBS (CAT, QUE, EGCG, and TA) at 200 μM for 48 h, in a shaking incubator, at 37°C. AC explants incubated only with PBS were used as controls. The treated explants were again incubated for 24 h in PBS to wash out free polyphenols, as their presence can inhibit collagenase. The explants were then incubated in collagenase at 37°C for 96 h. The ratio of collagen (in cartilage explants) to collagenase was maintained at 50:1 (w/w), and the reaction was buffered at pH 7.4 with 0.1-M Tris-HCl and 0.05-M CaCl_2_. After 96 h, the reaction was stopped, and the mixture was centrifuged for 15 min at 10,000 rpm. The supernatant was analyzed for soluble collagen and GAGs using the Woessner method[35] and DMMB dye[33, 34] respectively, and the percentage release thereof was calculated using the Eqs 1 and 2 given below.

$$\%\;\text{Collagen}\;\text{degraded}\;=\;\frac{\text{Amount}\;\text{of}\;\text{collagen}\;\text{(i.e}\;\text{amount}\;\text{of}\;\text{hydroxy}\;\text{proline}\;X\;\text{7.4)}\;\text{released}}{\text{Amount}\;\text{of}\;\text{total}\;\text{collagen}\;\text{estimated}\;\text{in}\;\text{cartilage}}\times 100$$ (1)

$$\%\;\text{GAG}\;\text{release}\;=\;\frac{\text{Amount}\;\text{of}\;\text{GAG}\;\text{released}}{\text{Amount}\;\text{of}\;\text{total}\;\text{GAG}\;\text{estimated}\;\text{in}\;\text{cartilage}}\times 100$$ (2)

### Physical stability of AC explants

To study the effects of the polyphenols on physical properties of cartilage, the compression strength of the cartilage with and without incubation in polyphenols was determined using a Brookfield CT3 10K Texture Analyzer (USA). AC explants 5 mm in diameter within a narrow weight range (18–22 mg) were obtained. The cartilage samples were separated into six groups (three samples per group). Five cartilage groups were incubated with or without 200 μM polyphenols (CAT, QUE, EGCG, and TA, respectively) at 37°C for 96 h. Another additional control group was stored at 4°C. The load required for 50% compression (i.e., half of its original thickness) of the incubated cartilage samples was determined. Prior to compression measurement, the cartilage samples were equilibrated with PBS pH 7.4 for 4 h at room temperature (25°C) and then placed directly below the probe in a stainless steel plate with a thin layer of PBS on the circular plate of the instrument. The probe was allowed to compress the cartilage with the target of 50% compression at a defined speed (0.1 mm/sec) to examine the unconfined compression properties [23, 39].

### *In vivo* analysis of the effect of polyphenol in protecting AC in collagen-induced arthritic rats

Forty-two female Wistar rats, 6–8 weeks old and weighing 130–180 g, were used to evaluate the effect of the intra-articular injection of polyphenols (EGCG and TA) in protecting AC under arthritic conditions. The animals were grouped for both prophylactic and therapeutic treatment conditions as shown in Table 1.

**Table 1:** Grouping of animals to evaluate the efficacy of prophylactic and therapeutic treatment of polyphenols.

Groups		Number of rats
Negative Control (NC)		6
**Prophylactic Treatment**	Positive Control (PPC)	6
	Treatment with EGCG (PE)	6
	Treatment with TA (PT)	6
**Therapeutic Treatment**	Positive Control (TPC)	6
	Treatment with EGCG (TE)	6
	Treatment with TA (TT)	6

The EGCG and TA solutions were prepared fresh each injection day by dissolving the desired weights in sterile saline under aseptic conditions. For each leg, a dose of 300 μg (i.e., 25 μl from 12 mg/ml stock solution) of EGCG or TA was injected intra-articularly in the tibiofemoral joints of the rats of the respective groups. Positive control (PPC & TPC) and NC were injected with saline.

### Collagen-induced arthritis

CIA was induced in all rats except the NCs (i.e., without disease) by the intradermal injection of an antigenic mixture in the back (dorsal part) and rear feet. Type II collagen (CII) from bovine AC was prepared and purified; the final concentration of 2 mg/ml was obtained by suitable dilution using 0.05 M acetic acid and was then stored at 4°C. Heat-killed *M*. *tuberculosis* (HKMtb) collected from the Tuberculosis Research Centre (TRC—Chennai, India) was processed as described previously [40]. On the day of CIA induction, HKMtb was emulsified in an ice bath with an equal volume of CII solution and CFA to obtain a final concentration of 2 mg/ml. Finally, the antigenic mixture (300 μl in six divided doses) was injected over four sites in the rats' backs and two at each foot on day 0 (induction injection) and similarly on day 7 (booster injection). The volumes of the rats' hind paws were examined periodically for arthritis development using a plethysmometer.

### Prophylactic treatment and induction of CIA

Through prophylactic treatment, we sought to ensure that the binding of polyphenols took place before the induction of arthritis; therefore, any significant protection of cartilage degradation would be direct proof of the binding of polyphenols to AC. Three prophylactic treatment groups with 6 rats per group (EGCG treatment [PE], TA treatment [PT], and sterile PBS—prophylactic positive control [PPC]) were used. Intra-articular injections of 25 μL (per leg) from 12 mg/ml stock of EGCG, TA, or PBS in five doses (on days -13, -10, -7, -4, and -1) were completed before the first arthritis induction immunization (on day 0).

### Induction of CIA and therapeutic treatment

For therapeutic treatment, five doses of polyphenols (TA and EGCG) or control PBS in 25 μL of volume were injected on days 19, 22, 25, 28, and 31 after immunization.

### Analysis of the articular cartilage

Both the prophylactic and therapeutic groups were sacrificed on day 43 of immunization, and the tibiofemoral joints of 3 rats in each group were fixed in 10% formalin. They were then decalcified and dehydrated and spliced sagitally into two halves (S1 Fig). Midsections of 3-μm thickness were obtained from embedded tissues on spliced side and stained with hematoxylin and eosin (H&E) and masson's trichrome [24]. Degradation (blind) scoring of both tibial and femoral end cartilage was carried out in six histological sections (i.e. six joints from 3 rats). Quantitative histomorphological scoring of cartilage degradation was as follows: Score 0 (normal cartilage), Score 1 (minimal damage), Score 2 (moderate damage), and Score 3 (maximum damage) [41]. An independent expert pathologist, without knowledge of the experimental groups did the scoring.

### Statistical analysis

The statistical analysis was performed with GraphPad Prism version 5. Statistical significance was defined as P-value ≤ 0.05. Data were analyzed by one-way ANOVA, RM-ANOVA, or the unpaired Student's *t*-test, as appropriate.

## Results

### Collagen, GAG, and water content

In RA and OA, the degradation of proteoglycans and collagen on the cartilage surface allows more water to penetrate and loosen the matrix, thereby affecting the load-bearing abilities of the cartilage. The presence of water, proteoglycans, and collagen is important in maintaining the physical properties of AC. The loss of proteoglycans will cause a change in the water content and subsequent loss of elasticity and resilience. The cartilage explants were analyzed for water content, which was in line with previous reports, at 70.21± 2.41% [42]. The total collagen content of the wet cartilage was 14.03 ± 0.25%, about 47% of the dry weight. The GAG content of the explants was found to be 5.24 ± 0.81% (on a wet-weight basis).

### Thermal stability of polyphenol-treated cartilage

**Fig 1:**
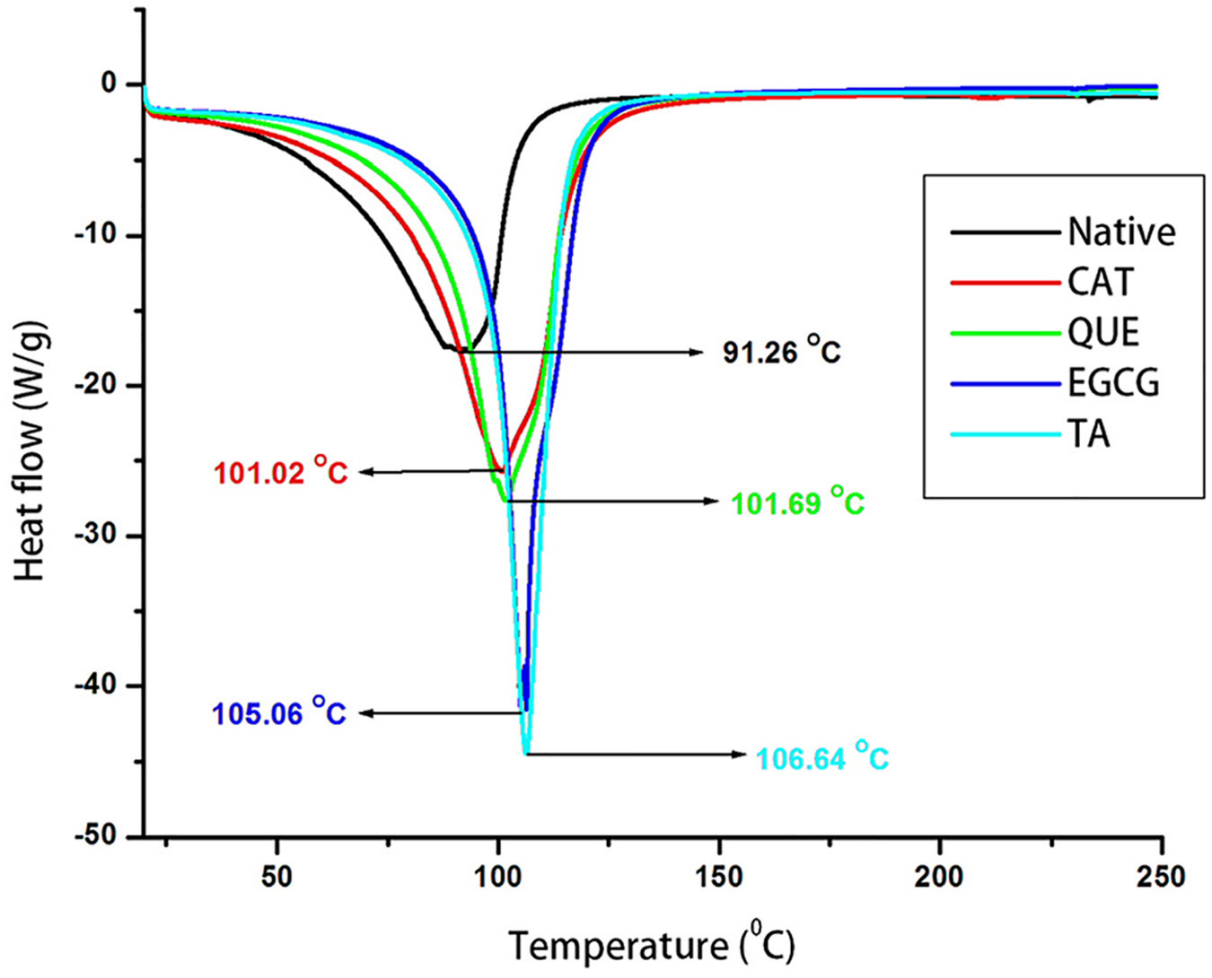
Differential scanning calorimetric (DSC) analysis. Thermograms of control (native) and polyphenol-treated (CAT, QUE, EGCG, and TA) bovine AC samples (representative picture)

Relatively little information is available on the thermal properties of mammalian hyaline cartilage, particularly the effect of drugs on the thermo-physiochemical properties of AC. Here, for the first time, bovine AC has been studied using DSC to evaluate the effect of polyphenols on the thermal stability of articular cartilage. DSC thermograms of native and polyphenol-treated AC are shown in Fig 1, and the denaturation temperature of cartilage is presented in Table 2. The polyphenol-treated cartilage showed increases in the thermal stability of collagen from 8–16°C with reference to native cartilage; EGCG and TA showed the maximum increases in the thermal stability of cartilage (12 and 16°C respectively), whereas QUE and CAT exhibited only 8 and 10°C increases in thermal stability respectively, with reference to native untreated cartilage. Enhancement in thermal stability indicates the binding of polyphenols with the collagen in the cartilage matrix, as well as collagen crosslinking. The aromatic rings of polyphenols could also be involved in hydrophobic association with aromatic side-chain functional groups of collagen type II[30, 43].

**Table 2:** Increase in thermal denaturation temperature of polyphenol-treated cartilage with reference to native untreated bovine cartilage determined using DSC.

Treatment	Increase in Thermal Denaturation Temperature^a^ (°C)
**Catechin**	10 ± 0
**Quercetin**	8.5 ± 1.5
**Epigallocatechin gallate**	12 ± 2
**Tannic acid**	17.5 ± 0.5

^a^ n = 2; experiments were performed on two independent sets of bovine AC.

### Enzymatic stability of cartilage explants

Polyphenol binding and crosslinking render cartilage resistant to enzymatic degradation. The percentages of collagen and GAG released from the cartilage explants after the enzymatic treatment are presented in Table 3. The untreated cartilage showed collagen degradation of about 72%, whereas the polyphenol-treated cartilage samples showed a significant level of protection from enzymatic collagen degradation. The EGCG, TA, and CAT treatments were more protective (i.e., statistically significant), showing only 24, 29, and 32% collagen degradation, respectively, against collagenase digestion. QUE showed lesser protective action against collagenolytic degradation. GAG release from the cartilage matrix after collagenase treatment was also lower (statistically significant) for cartilage pre-treated with EGCG (9.1%) and TA (8.9%) than the control which was 18.8%; whereas, cartilage treated with CAT and QUE showed 13.1 and 12.8% GAG release, respectively.

**Table 3:** Percentage of collagen degradation and release of GAGs from bovine AC (with and without polyphenols) treated with collagenase.

Polyphenol (200 μM)	% Degradation of collagen (from AC)^a^	% Release of GAGs^a^
**Control(PBS medium)**	72.5 ± 13.6	18.86 ± 0.60
**Catechin**	32.1 ± 6.0*	13.18 ± 1.43
**Quercetin**	42.7 ± 4.9	12.89 ± 1.19
**Epigallocatechin gallate**	24.6 ± 1.3*	9.10 ± 1.59*
**Tannic acid**	29.0 ± 3.4*	8.96 ± 1.07*

^a^Data were analyzed using one-way ANOVA with Bonferroni post hoc test. The EGCG (P<0.01), TA (P<0.05), and CAT (P<0.05) treated groups showed significantly reduced percent degradation of collagen compared to controls. Similarly, the percent release of GAG in the EGCG- (P<0.01) and TA-treated (P<0.01) groups had significantly reduced (P-value: 0.0268) compared to controls but no statistical significance was observed in CAT and QUE. The values are represented as Mean ± SEM, n = 3.

(* indicates significant difference in comparison to control, P<0.05.)

### Physical stability of AC (compression analysis)

**Fig 2:**
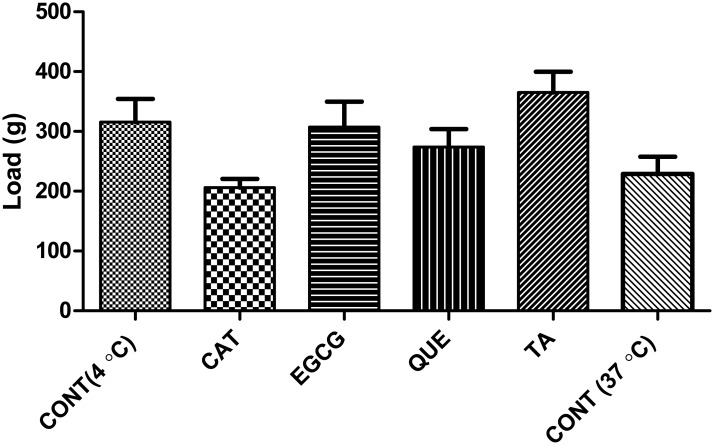
Compression analysis of AC. Compressive load at 50% compression of AC incubated 4 days without and with polyphenols 200-μM (CAT, QUE, EGCG, TA) prepared using PBS; figure shows day-4 results; no significant difference (P>0.05) was observed between the control and polyphenol-treated samples; values are represented in Mean ± SEM, n = 3.

Load-bearing ability is an important physical property of the AC; however, the cushioning property of cartilage is lost if the collagen and GAG are degraded. The effect of polyphenolic interactions may alter the compressive properties of the cartilage. To determine the changes in compressive properties, the compression load of AC samples with and without polyphenol treatment were analyzed; cartilage samples incubated for 96 h are presented in Fig 2. The control cartilage incubated in PBS for 96 h at 4°C and 37°C exhibited a mean load of 315.8 and 228.8 g for 50% compression of the AC. The mean values of polyphenol-treated ACs were not statistically different from the control values shows that there is no alteration in the mechanical properties of polyphenol treated AC samples.

### Effect of intra-articular injections of polyphenol (EGCG or TA) in AC protection of CIA rats

*In vitro* studies showed that the treatment of cartilage with polyphenols stabilizes the cartilage enzymatically and thermally, and does not significantly alter the mechanical properties. Because of the poor bioavailability of polyphenols in circulation, particularly in synovial fluids, it is likely that limited observations have been made concerning its salutary effect on AC. We hypothesize that polyphenols injected intra-articularly will interact with type II collagen of AC and stabilize it against degradation by MMPs. Furthermore, the more efficient (in the thermal and enzymatic stabilization of AC) polyphenols (EGCG and TA) were selected for the *in vivo* studies of the protective effect on cartilage degradation using CIA rat models. We evaluated two strategies: prophylactic and therapeutic intra-articular injection in a CIA rat model.

### Prophylactic treatment

**Fig 3:**
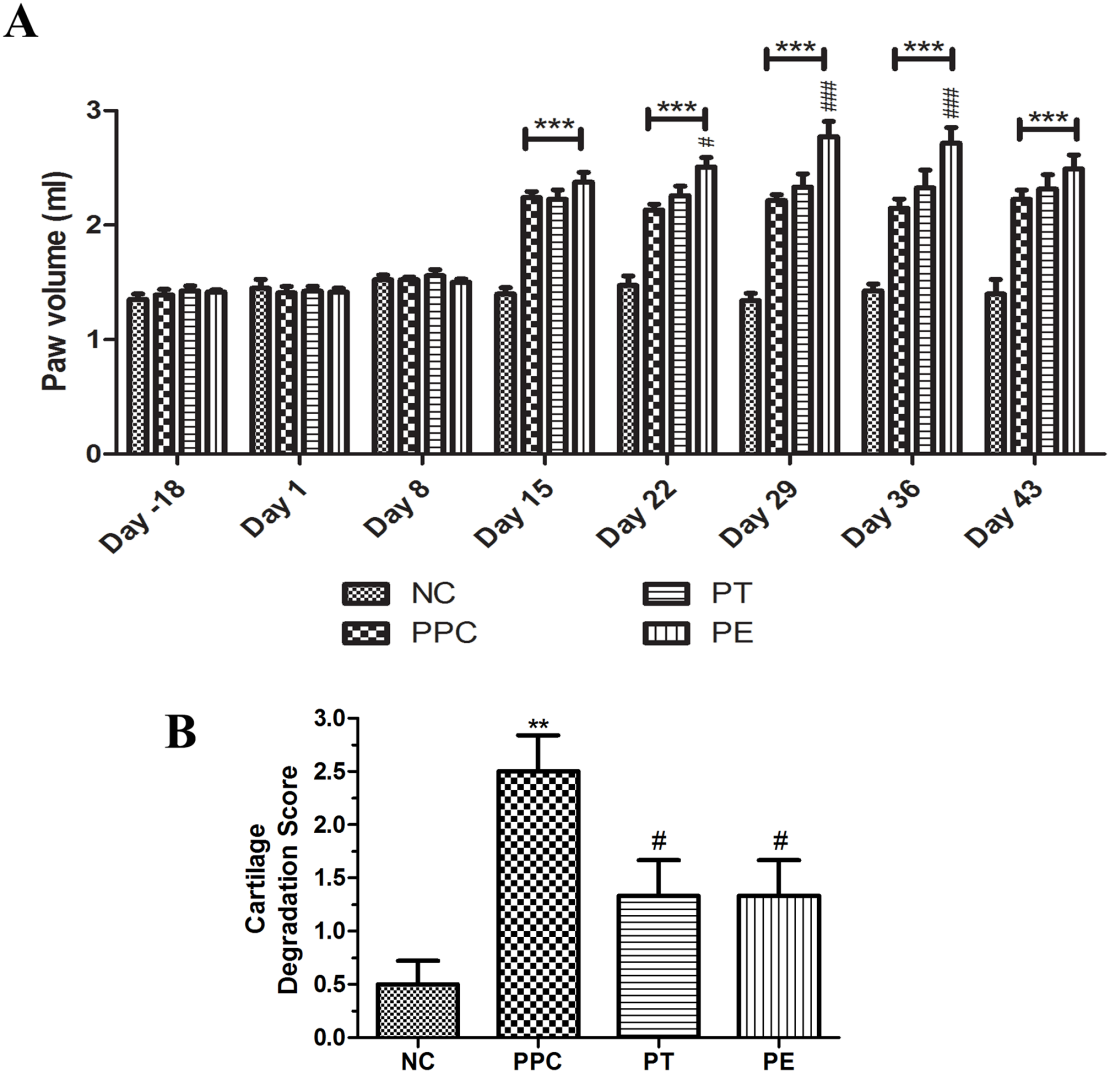
Paw volume and cartilage degradation scoring of prophylactically treated groups. (A) Paw volume changes in prophylactically treated groups from day -18 to 43 (values analyzed using RM- ANOVA with Graph Pad prism); "***" indicates significant difference compared to NC (P<0.001); "#" indicates significant difference compared to PPC (### represents P<0.001 and # represents P<0.05); values represented in Mean ± SEM, n = 12. (B) Cartilage degradation scores from histomorphological sections of prophylactically treated groups; "**" indicates significant difference from NC (P<0.01); statistically significant differences between PPC and PT or PE groups indicated as "#" (P<0.05); values represented in Mean ± SEM, n = 6.

In the prophylactic treatment group, intra-articular injections of EGCG, TA, and PBS in five doses were completed before arthritis induction. The development of CIA was monitored by paw volume measurements. As shown in Fig 3A, paw volume was unchanged until day 8 in immunized rats. On day 15, we observed a significant (P<0.001, n = 6) increase in paw volume in PPC, PT, and PE compared to NC, indicating the onset of CIA in immunized rats. There was no significant difference in paw volume at the onset of CIA between the PPC and the PT or PE groups. Beginning on day 22, the PE rats showed significant increases in paw volume until day 36 compared to the PPC rats, but this was not observed in the PT groups. A significant maximum increase in paw volume was observed on day 29 (i.e., increase to 2.78 ml versus 2.21 ml in the positive controls). These observations indicate that the prophylactic treatments of polyphenols EGCG and TA did not alleviate inflammation in the paws of immunized rats.

**Fig 4:**
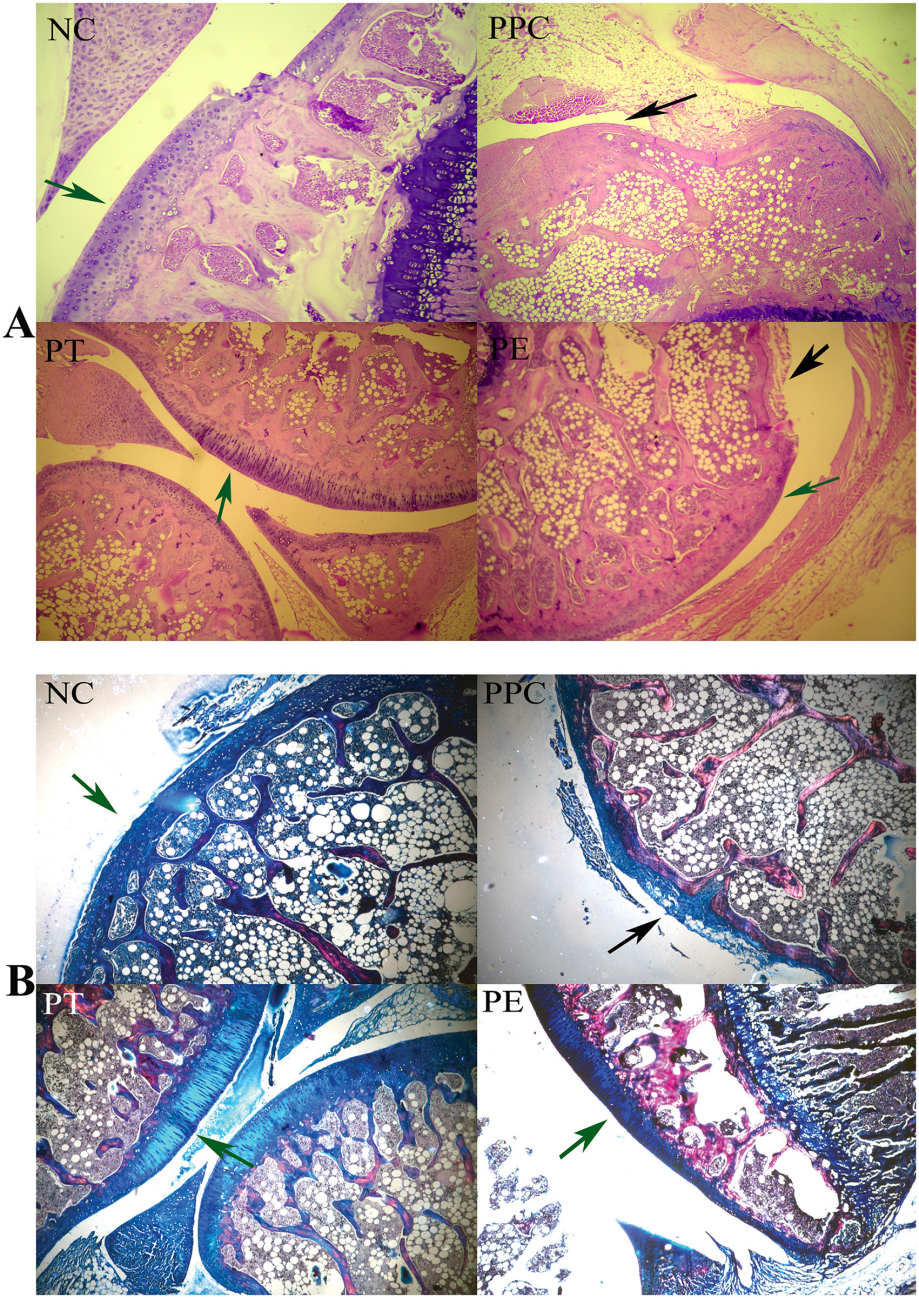
Effect of prophylactic treatment of polyphenols on collagen-induced arthritis (CIA). (A) Hematoxylin and Eosin-stained sections and (B) Masson's Trichrome stained sections of tibiofemoral joints of rats; NC indicates Negative Control (i.e., normal joint without induction and treatment); remaining illustrations are from CIA joints: PPC—Prophylactic Positive Control; PE—Prophylactic EGCG; and PT—Prophylactic TA; (black arrow = disintegrated cartilage; green arrows = intact cartilage)

The evaluation of histological sections of joints from various groups was done blindly for cartilage damage and synovial inflammation. The synovial membrane and cartilage in the NC group was normal. PPC exhibited synovial adhesions and fibrous fatty tissue, and the synovial membrane was inflamed and hyperplasic (Fig 4A), confirming the induction of CIA. The synovial membranes of PT showed more inflammation than PE, whereas the latter group had few eosinophilic infiltrations. The dark blue stain (by Masson's trichrome staining) in the histological sections at the articulating surface indicates the type II collagen matrix of AC (Fig 4B). In the NC, PT, and PE sections, the collagen was intact (green arrows), whereas the PPC showed irregular collagen matrix loss (black arrows). Histomorphological scoring levels of the cartilage degradation are presented in Fig 3B. As shown, the combined scoring (of three joints) for cartilage degradation in PT and PE was significantly lower than in PPC (P<0.05). In addition, AC in PT showed some superficial surface irregularities and rice bodies in the joint space (Fig 4A and 4B).

### Therapeutic treatment

**Fig 5:**
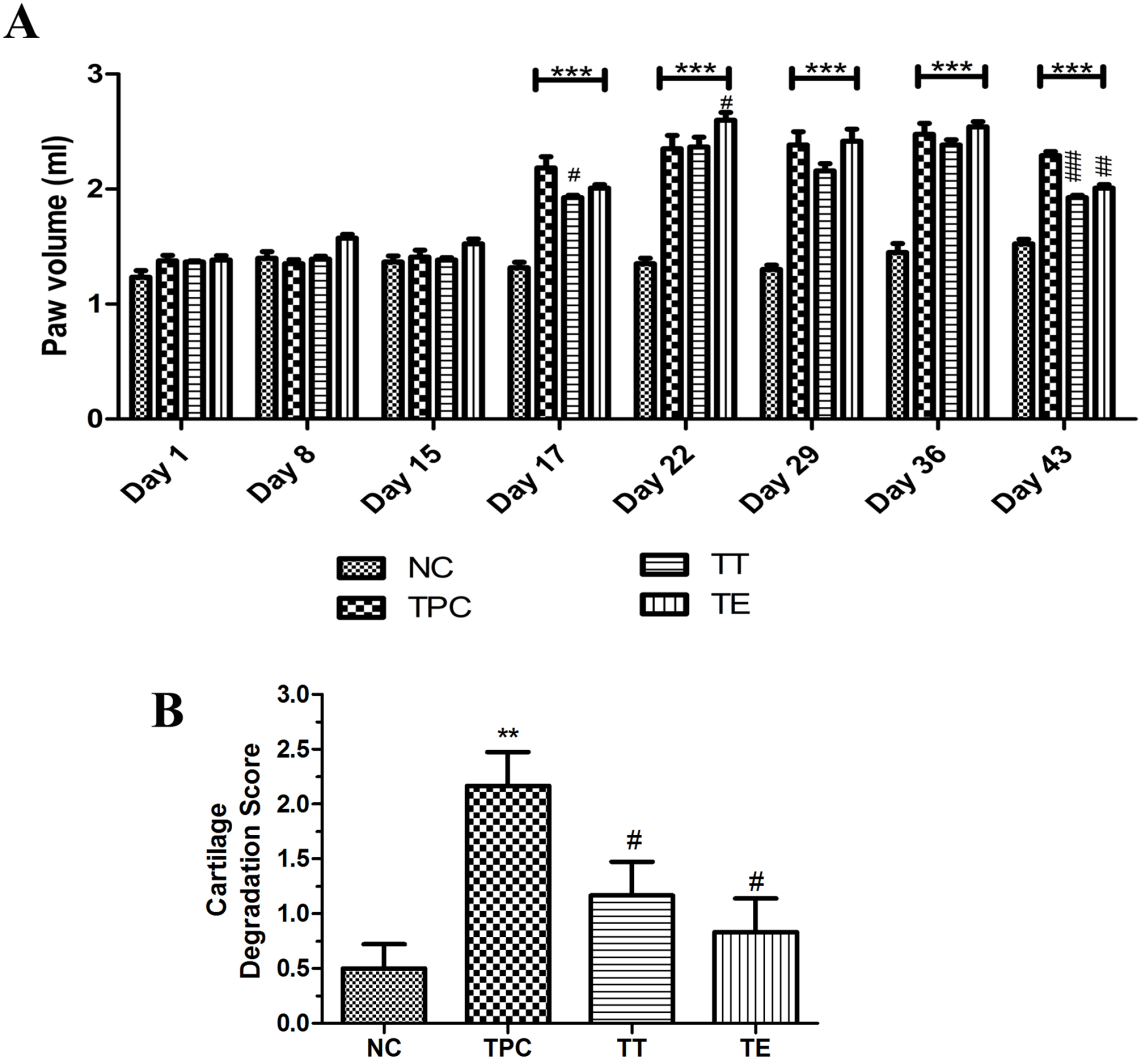
Paw volume and cartilage degradation scoring of therapeutically treated groups. (A) Paw volume changes in therapeutically treated rat groups from day 1–43 (values analyzed using RM-ANOVA with Graph Pad prism; "***" indicates significant difference compared to negative control (NC) (P<0.001); "#" indicates significant difference compared to therapeutic positive control (TPC) (### represents P<0.001, ## represents P<0.01 and # represents P<0.05); values represented in Mean ± SEM, n = 12. (B) Cartilage degradation scores from histomorphological sections of therapeutically treated groups; "**" indicates significant difference compared to NC (P<0.01); statistically significant differences between TPC and TT or TE groups indicated as "#" (P<0.05); values represented in Mean ± SEM, n = 6.

In the therapeutic groups, intra-articular injections of EGCG, TA, and PBS in five doses were completed after arthritis induction. As shown in Fig 5A, the paw volume (indicator of CIA onset) began to change on day 17, confirming significant induction of CIA (P<0.001, n = 6) in the TPC, TT, and TE groups compared to NC. Early on, paw volume was not significantly different among these groups. In the case of the therapeutic EGCG treatment, paw volume significantly increased on day 22 (P<0.05) but on day 43 (final) it had dropped significantly (P<0.01) to a volume of 2.00 ml compared to positive controls (2.29 ml). On day 43, the TT group also showed a significant (P<0.001) decrease in paw volume compared to TPC (i.e., 1.93 ml for TA and 2.29 ml for positive controls) (Fig 5A). There was no significant difference between TA and EGCG in reduction of paw inflammation. These observations indicate that therapeutic treatment with polyphenols EGCG and TA did not result in any significant alteration of paw inflammation.

**Fig 6:**
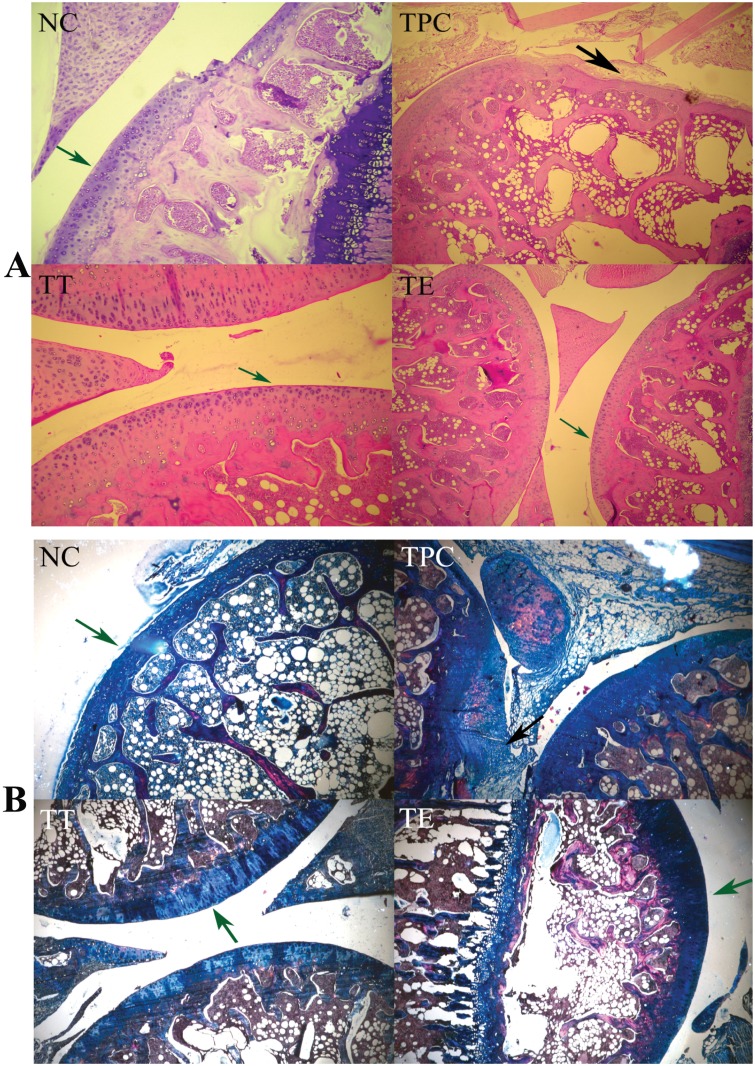
Effect of therapeutic treatment of polyphenols on collagen-induced arthritis (CIA). (A) Hematoxylin and Eosin-stained sections and (B) Masson's Trichrome stained sections of tibiofemoral joints of rats. NC indicates Negative Control normal joint (without induction and treatment); remaining illustrations are from collagen-induced arthritis joints; TPC—Therapeutic Positive Control; TE—Therapeutic EGCG; TT—Therapeutic TA; black arrow = disintegrated cartilage; green arrows = intact cartilage)

The combined histomorphological cartilage damage scores (of six joints) in the therapeutic groups of TA-treated (TT) and EGCG-treated (TE) cartilage were significantly lower (P<0.05) than that of TPC (Fig 5B). The cartilage damage scoring of TE was slightly lower than that of TT but did not reach statistical significance. As shown in H&E histological sections, the cartilage damage was very mild to negligible in most TT and TE samples (Fig 6A and 6B). Upon further observation using Masson's trichrome, the cartilage damage was minimal and the cartilage appeared intact in the stained sections (Fig 6B) of the therapeutically treated joints in groups TT and TE. However, the cartilage in group TPC (as shown) was profusely degraded (black arrows). Cartilage in normal controls was intact (Fig 6B, green arrows). Synovial inflammation was found in both the TA- and EGCG-treated therapeutic groups. The severity of synovial inflammation in both TT and TE was lower than in TPC.

## Discussion

**Fig 7:**
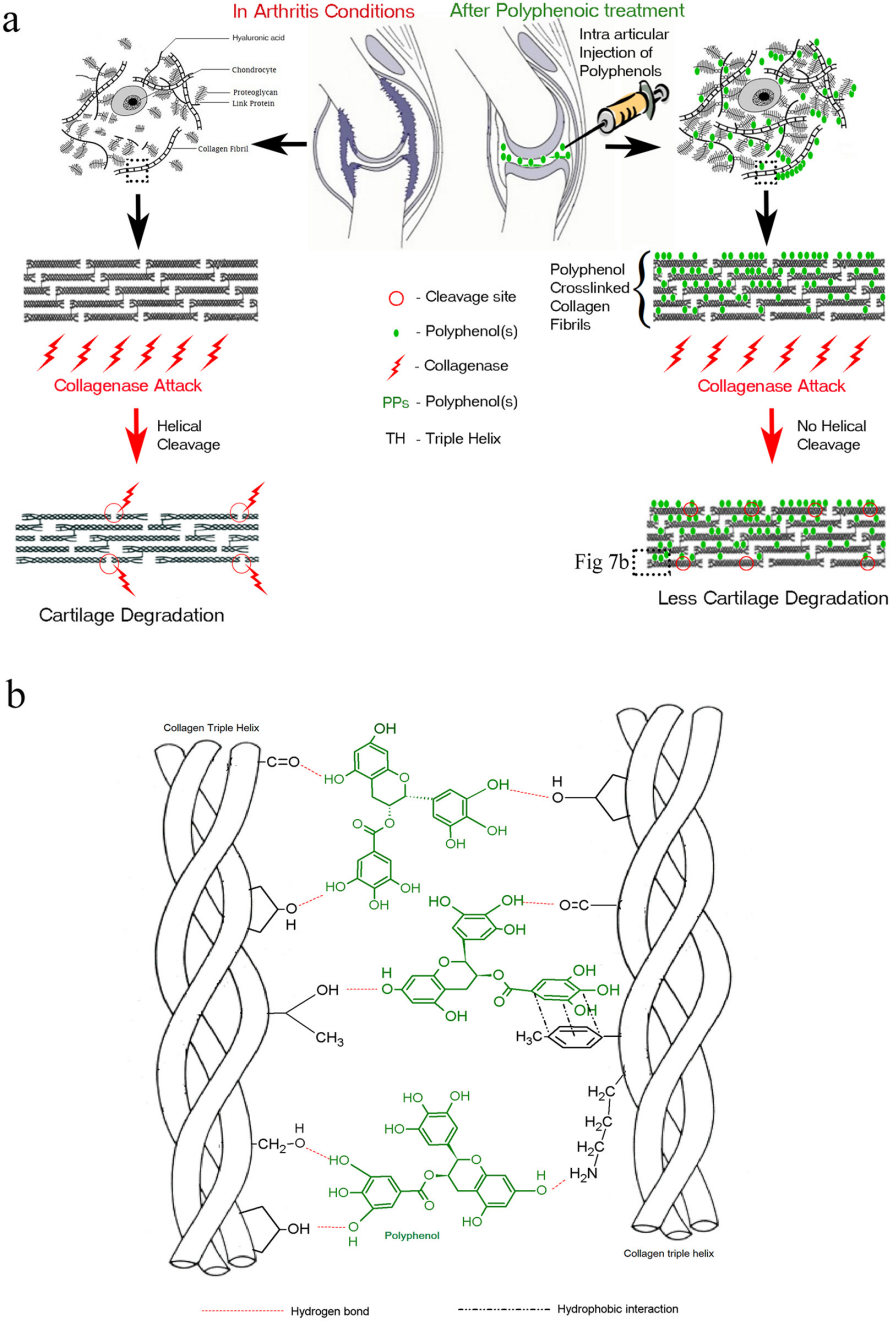
**Polyphenolic treatment to articular cartilage protects collagen against degradation** a) Mechanistic pathway of polyphenols role in protecting articular cartilage b) Possible Hydrogen and hydrophobic interaction of polyphenols with type II collagen triple helical molecules in the cartilage matrix.

Supra-molecular assemblies of type II collagen (CII) are similar to those of type I, forming fibrillar structures. AC, composed predominantly of CII, is a porous matrix that facilitates the diffusion of small molecules through the porous cartilage matrix, which could crosslink with the side-chain functional groups of collagen, as shown in Fig 7. We show for the first time that treatment with polyphenols increases the thermal stability of AC. Of the various polyphenols tested, EGCG and TA had greater effects on thermal stability. Collagen is an inside-out protein where the side-chain functional groups are projected outward and these can react with multiple hydroxyl functional groups of polyphenols resulting in multiple hydrogen-bonded interactions with collagen, thereby conferring stability to the cartilage matrix (Fig 7B). Plant polyphenolic molecules can stabilize type I collagenous matrices through hydrogen bonding and hydrophobic interactions [44, 45]. Schlebusch and Kern [46] studied the possible stabilizing effects of catechin on collagen for vascular tissue stabilization. Similarly various studies reports stabilization of collagenous tissues through natural crosslinking agents. Cardiovascular tissues [47–52], intestinal mucosa[53], corneas[54, 55], tendons[56], dentin[57], cartilage[58] and other collagenous scaffolds[59] has been studied with crosslinkers like glutaraldehyde, tannic acid, pentagalloyl glucose, genipin, procyanidins and lysyl oxidase etc. Plant polyphenols are found to be equivalent and advantageous in stabilizing the tissues compared to the chemical crosslinking agent glutaraldehyde which are found to be irreversible, cytotoxic and produces calcification[50, 51].

With the evidence that polyphenols bind to AC, we studied their efficacy in protecting cartilage from enzymatic degradation. EGCG and TA treatments showed greater effects in protecting the cartilage from enzymatic damage, whereas QUE and CAT had lesser effects (Table 3). This is consistent with increases in the thermal stability of AC caused by EGCG and TA. In addition to the collagenolytic degradation of AC, there was a significant release of GAGs from the enzyme-treated AC, which must have been due to the release of GAGs associated with the fragments of collagen fibrils digested from the cartilage. Interestingly, both TA and EGCG treatment exhibited higher efficacy in stabilizing cartilage for collagen degradation and GAG release than did either QUE or CAT. This could be due to the ability of EGCG and TA to form better crosslinking with collagen and other matrix components.

Recently, a study to improve collagen-based biomaterial used for skin anti-aging was conducted using polyphenols, revealing that they block the site at which collagenase acts upon collagen [60]. Previous studies have also reported the collagenase inhibitory effects of polyphenols [60, 61]. In our studies, the polyphenol-treated cartilage was thoroughly washed to leach out any free or weakly bound polyphenols and hence eliminating the possibility of having free polyphenols to inhibit collagenase. Therefore, the resistance to collagenolytic degradation was predominantly due to increased crosslinks between polyphenols and CII in the cartilage.

The presence of water, proteoglycans, and collagen is important in maintaining the compressive properties of AC. The loss of proteoglycans will cause a change in the water content and subsequent loss of elasticity and resilience, and the cushioning property will be lost if the collagen and GAG are degraded in cartilage. Therefore, it was important to study the effect of polyphenols on the compressive strength of cartilage. As shown, polyphenol-treated cartilage explants showed less deterioration in load-bearing ability than controls (Fig 2). The change in load after polyphenol treatment was not more than 10% compared to the change in untreated cartilage (controls). This shows that polyphenolic treatment did not alter the mechanical (compression) properties of cartilage compared to the untreated cartilage explants. Similarly, Sung HW et al reported that genipin and glutaraldehyde treatment to porcine aortic valves did not seem to have significant difference in altering the mechanical properties. Glutaraldehyde treatment showed an increased denaturation temperature (~ 22°C) than that of Genipin (~15°C) compared to that of untreated tissues[62]. Recently, Satyam et al reported, that treatment of genipin with collagen scaffold was found to improve enzymatic and thermal stability (by ~ 24°C) than that of control and produces significant change in the mechanical properties[59].

### Polyphenols protecting articular cartilage

To enable the polyphenols to be efficient, the intra articular route of injection was selected, which will provide the advantage of direct drug administration but can be subjected to rapid clearance (t_1/2_ of 0.1–6 h)[63]. Additional advantage is that biochemically intact polyphenols without being subjected to metabolic changes in the intestinal or hepatic systems would be delivered in the joints. We hypothesize that polyphenols injected intra-articularly will interact with type II collagen and stabilize cartilage against degradation, as illustrated in Fig 7B. The more efficient polyphenols (in the enzymatic stabilization of AC), EGCG and TA were selected for the *in vivo* studies using CIA rat models.

The onset of CIA was clearly established with increases in paw volumes in both the prophylactic and therapeutic groups (Figs 3A and 4A). The lack of change in paw volume in the prophylactic group (Fig 3A) is a clear indication that polyphenols have little influence on the time of onset of arthritis or the maintenance of the inflammation level as measured by paw volume; whereas, in the case of the therapeutic groups, there was again no delay in the onset of arthritis or the maintenance of inflammation, except for some reduction in paw volume at the end of the experiment (day 43) (Fig 4A) compared to the positive controls. In all cases, the positive controls (immunized) had significant inflammation compared to the un-immunized (PBS only) controls. Against this backdrop, the prophylactic intra-articular administration of EGCG or TA showed significant (P<0.05) protective effects on the degree of cartilage damage as measured by histomorphological scoring compared to the positive controls (Figs 3B and 5B). This indicates that AC in prophylactic polyphenol-injected joints becomes resistant to the subsequent induction of inflammation-induced degradation.

In the therapeutic polyphenol group, there was some reduction in paw volume, indicating a decrease in the degree of inflammation but only during the latter part of CIA, as compared to paw volume in positive controls. This may be consistent with the known anti-inflammatory effects of polyphenols. The therapeutic groups also showed the significant benefit of intra-articular injections of polyphenols in protecting cartilage from degradation during CIA (Figs 4B and6B). These observations are statistically significant (P<0.05). EGCG showed a better cartilage-protecting effect than TA. Together this indicates that AC in therapeutic polyphenol-injected joints becomes resistant to concurrent inflammation-induced degradation compared to subsequent inflammation in the prophylactic groups. Previously, catechin (20 μM) was found to inhibit the degradation of bovine nasal and AC explants by inhibiting the chondrocyte catabolic response [15]. In another study, EGCG (100 and 200 μM) was effective in inhibiting the IL-1β-induced production of matrix-degrading enzymes [34]. Recently, curcumin (1–25 μM) and quercetin (10–50 μM) also inhibited the matrix-degrading enzymes[64]. It is possible the protective effect on cartilage in the therapeutic groups could have been caused by the direct inhibition of MMPs. However, polyphenols in synovial fluid would have been rapidly cleared and metabolized, and little would have remained to inhibit the gradual degrading process of MMPs on the cartilage. Another explanation for the protective effect on cartilage could be a reduction in the degree of inflammation in the joints, but this was observed only in the latter part of CIA and was not confirmed by histopathological observation of inflammation in the synovium. Based on our *in vitro* data and the observations in the prophylactic polyphenol treatment groups, we predominantly credit the binding of polyphenols to AC as the likely protective factor against cartilage degradation in the therapeutic groups.

Human collagen type II in the AC matrix is a long-lived protein with an estimated half-life of >117 years [65]. Collagen maturation involves extensive crosslinking, a strategy used for cartilage maintenance throughout the lifespan of most humans. Based on conventional wisdom of vegetable tanning, we have hypothesized that polyphenols could be used to crosslink cartilage collagen type II, rendering it resistant to degradation, as shown in schematic form in Fig 7. Intra-articular injections of polyphenols prevented cartilage degradation amid the milieu of inflamed joints. Further research is warranted to study the effects of polyphenols injections into the joint cavity and also establishing its role in an osteoarthritis models. In summary, we suggest a unique novel role for intra-articular injections of polyphenols in the therapeutics of cartilage degradation.

## Supporting Information

S1 Fig Schematic representation of the sampling (sectioning) of rat joint for histolgical analysis of cartilage. (TIF)

## References

[1] Aly MN. Intra-articular drug delivery: a fast growing approach. Recent patents on drug delivery & formulation. 2008;2(3):231–7. . 19075910 10.2174/187221108786241651

[2] Ofek G, Revell CM, Hu JC, Allison DD, Grande-Allen KJ, Athanasiou KA. Matrix development in self-assembly of articular cartilage. Plos One. 2008;3(7):e2795 10.1371/journal.pone.0002795 18665220 PMC2464773

[3] Sophia Fox AJ, Bedi A, Rodeo SA. The basic science of articular cartilage: structure, composition, and function. Sports health. 2009;1(6):461–8. . 23015907 10.1177/1941738109350438 PMC3445147

[4] Mort JS, Billington CJ. Articular cartilage and changes in arthritis: matrix degradation. Arthritis research. 2001;3(6):337–41. . 11714387 10.1186/ar325 PMC128908

[5] Buckwalter JA, Mankin HJ. Articular cartilage: degeneration and osteoarthritis, repair, regeneration, and transplantation. Instructional course lectures. 1998;47:487–504. . 9571450

[6] Stanton H, Rogerson FM, East CJ, Golub SB, Lawlor KE, Meeker CT, et al ADAMTS5 is the major aggrecanase in mouse cartilage in vivo and in vitro. Nature. 2005;434(7033):648–52. 15800625 10.1038/nature03417

[7] Ulrich-Vinther M, Maloney MD, Schwarz EM, Rosier R, O'Keefe RJ. Articular cartilage biology. The Journal of the American Academy of Orthopaedic Surgeons. 2003;11(6):421–30. . 14686827 10.5435/00124635-200311000-00006

[8] Tyyni A, Karlsson J. Biological treatment of joint cartilage damage. Scandinavian journal of medicine & science in sports. 2000;10(5):249–65. 10.1136/bjsports-2015-094781 11001393

[9] Athens AA, Makris EA, Hu JC. Induced collagen cross-links enhance cartilage integration. PloS one. 2013;8(4):e60719 10.1371/journal.pone.0060719 23593295 PMC3617163

[10] Tiku ML, Narla H, Jain M, Yalamanchili P. Glucosamine prevents in vitro collagen degradation in chondrocytes by inhibiting advanced lipoxidation reactions and protein oxidation. Arthritis research & therapy. 2007;9(4):R76 . 17686167 10.1186/ar2274 PMC2206377

[11] Pandey KB, Rizvi SI. Plant polyphenols as dietary antioxidants in human health and disease. Oxidative Medicine and Cellular Longevity. 2009;2(5):270–8. 10.4161/oxim.2.5.9498 20716914 PMC2835915

[12] Manach C, Scalbert A, Morand C, Remesy C, Jimenez L. Polyphenols: food sources and bioavailability. The American journal of clinical nutrition. 2004;79(5):727–47. . 15113710 10.1093/ajcn/79.5.727

[13] Mohan A, Narayanan S, Sethuraman S, Maheswari Krishnan U. Combinations of plant polyphenols & anti-cancer molecules: a novel treatment strategy for cancer chemotherapy. Anti-Cancer Agents in Medicinal Chemistry (Formerly Current Medicinal Chemistry-Anti-Cancer Agents). 2013;13(2):281–95. 22721388 10.2174/1871520611313020015

[14] Kona FR, Shen M, Chen D, Chan TH, Dou QP. Discovery of Polyphenol-Based Drugs for Cancer Prevention and Treatment: The Tumor Proteasome as a Novel Target. Recent Advances in Polyphenol Research. 2014;4:209.

[15] Adcocks C, Collin P, Buttle DJ. Catechins from green tea (Camellia sinensis) inhibit bovine and human cartilage proteoglycan and type II collagen degradation in vitro. The Journal of nutrition. 2002;132(3):341–6. . 11880552 10.1093/jn/132.3.341

[16] Yang CS, Hong J. Prevention of chronic diseases by tea: possible mechanisms and human relevance. Annual review of nutrition. 2013;33:161–81. 10.1146/annurev-nutr-071811-150717 23642203

[17] Cheng DM, Pogrebnyak N, Kuhn P, Krueger CG, Johnson WD, Raskin I. Development and Phytochemical Characterization of High Polyphenol Red Lettuce with Anti-Diabetic Properties. Plos One. 2014;9(3):e91571 10.1371/journal.pone.0091571 24637790 PMC3956610

[18] Htay HH, Kapoor MP. Green Tea Polyphenols in Cardiovascular Diseases. Green Tea Polyphenols: Nutraceuticals of Modern Life. 2013:139.

[19] Liu X, Kim JK, Li Y, Li J, Liu F, Chen X. Tannic acid stimulates glucose transport and inhibits adipocyte differentiation in 3T3-L1 cells. The Journal of nutrition. 2005;135(2):165–71. . 15671208 10.1093/jn/135.2.165

[20] Chang TL, Wang CH. Combination of quercetin and tannic acid in inhibiting 26S proteasome affects S5a and 20S expression, and accumulation of ubiquitin resulted in apoptosis in cancer chemoprevention. Biological chemistry. 2013;394(4):561–75. 10.1515/hsz-2012-0277 23241588

[21] Chen X, Beutler JA, McCloud TG, Loehfelm A, Yang L, Dong HF, et al Tannic acid is an inhibitor of CXCL12 (SDF-1alpha)/CXCR4 with antiangiogenic activity. Clin Cancer Res. 2003;9(8):3115–23. . 12912963

[22] Clutterbuck AL, Mobasheri A, Shakibaei M, Allaway D, Harris P. Interleukin-1beta-induced extracellular matrix degradation and glycosaminoglycan release is inhibited by curcumin in an explant model of cartilage inflammation. Annals of the New York Academy of Sciences. 2009;1171:428–35. 10.1111/j.1749-6632.2009.04687.x 19723086

[23] Ahmed S, Silverman MD, Marotte H, Kwan K, Matuszczak N, Koch AE. Down-regulation of myeloid cell leukemia 1 by epigallocatechin-3-gallate sensitizes rheumatoid arthritis synovial fibroblasts to tumor necrosis factor alpha-induced apoptosis. Arthritis and rheumatism. 2009;60(5):1282–93. 10.1002/art.24488 19404960 PMC2917979

[24] Morinobu A, Biao W, Tanaka S, Horiuchi M, Jun L, Tsuji G, et al (-)-Epigallocatechin-3-gallate suppresses osteoclast differentiation and ameliorates experimental arthritis in mice. Arthritis and rheumatism. 2008;58(7):2012–8. 10.1002/art.23594 18576345

[25] Yun HJ, Yoo WH, Han MK, Lee YR, Kim JS, Lee SI. Epigallocatechin-3-gallate suppresses TNF-alpha-induced production of MMP-1 and -3 in rheumatoid arthritis synovial fibroblasts. Rheumatology international. 2008;29(1):23–9. 10.1007/s00296-008-0597-5 18496696

[26] Shen CL, Smith BJ, Lo DF, Chyu MC, Dunn DM, Chen CH, et al Dietary polyphenols and mechanisms of osteoarthritis. The Journal of nutritional biochemistry. 2012;23(11):1367–77. 10.1016/j.jnutbio.2012.04.001 22832078

[27] Henrotin Y, Clutterbuck AL, Allaway D, Lodwig EM, Harris P, Mathy-Hartert M, et al Biological actions of curcumin on articular chondrocytes. Osteoarthritis and cartilage / OARS, Osteoarthritis Research Society. 2010;18(2):141–9. 10.1016/j.joca.2009.10.002 19836480

[28] Jean-Gilles D, Li L, Ma H, Yuan T, Chichester CO 3rd, Seeram NP. Anti-inflammatory effects of polyphenolic-enriched red raspberry extract in an antigen-induced arthritis rat model. Journal of agricultural and food chemistry. 2012;60(23):5755–62. 10.1021/jf203456w 22111586 PMC3306488

[29] Charlton AJ, Baxter NJ, Khan ML, Moir AJG, Haslam E, Davies AP, et al Polyphenol/Peptide Binding and Precipitation. Journal of agricultural and food chemistry. 2002;50(6):1593–601. 10.1021/jf010897z 11879042

[30] Baxter NJ, Lilley TH, Haslam E, Williamson MP. Multiple Interactions between Polyphenols and a Salivary Proline-Rich Protein Repeat Result in Complexation and Precipitation. Biochemistry. 1997;36(18):5566–77. 10.1021/bi9700328 9154941

[31] Madhan B, Muralidharan C, Jayakumar R. Study on the stabilisation of collagen with vegetable tannins in the presence of acrylic polymer. Biomaterials. 2002;23(14):2841–7. . 12069323 10.1016/s0142-9612(01)00410-0

[32] Madhan B, Thanikaivelan P, Subramanian V, Raghava Rao J, Unni Nair B, Ramasami T. Molecular mechanics and dynamics studies on the interaction of gallic acid with collagen-like peptides. Chemical Physics Letters. 2001;346(3â€“4):334–40.

[33] Pearson W, Fletcher RS, Kott LS, Hurtig MB. Protection against LPS-induced cartilage inflammation and degradation provided by a biological extract of Mentha spicata. BMC complementary and alternative medicine. 2010;10:19 10.1186/1472-6882-10-19 20459798 PMC2874512

[34] Ahmed S, Wang N, Lalonde M, Goldberg VM, Haqqi TM. Green tea polyphenol epigallocatechin-3-gallate (EGCG) differentially inhibits interleukin-1 beta-induced expression of matrix metalloproteinase-1 and -13 in human chondrocytes. The Journal of pharmacology and experimental therapeutics. 2004;308(2):767–73. . 14600251 10.1124/jpet.103.059220

[35] Woessner JF Jr. The determination of hydroxyproline in tissue and protein samples containing small proportions of this imino acid. Archives of biochemistry and biophysics. 1961;93:440–7. . 13786180 10.1016/0003-9861(61)90291-0

[36] Csotye J, Aigner Z, Sohar G, Szabo-Revesz P, Toth K. Calorimetric properties of degenerative human shoulder joint hyaline cartilage. Journal of Thermal Analysis and Calorimetry. 2009;95(3):805–8.

[37] Naumov I, Wiegand N, Patczai B, Vámhidy L, Lőrinczy D. Differential scanning calorimetric examination of the human hyaline cartilage of the femoral head after femoral neck fracture. Journal of Thermal Analysis and Calorimetry. 2012;108(1):59–65. 10.1007/s10973-011-1532-7

[38] Than P, Kereskai L. Thermal analysis of the osteoarthritic human hyaline cartilage. Journal of Thermal Analysis and Calorimetry. 2005;82(1):213–6.

[39] Barker MK, Seedhom BB. The relationship of the compressive modulus of articular cartilage with its deformation response to cyclic loading: does cartilage optimize its modulus so as to minimize the strains arising in it due to the prevalent loading regime? Rheumatology (Oxford, England). 2001;40(3):274–84. . 11285374 10.1093/rheumatology/40.3.274

[40] Ganesan K, Selvam R, Abhirami R, Raju KV, Manohar BM, Puvanakrishnan R. Gender differences and protective effects of testosterone in collagen induced arthritis in rats. Rheumatology international. 2008;28(4):345–53. . 17763851 10.1007/s00296-007-0446-y

[41] Svelander L, Erlandsson-Harris H, Astner L, Grabowska U, Klareskog L, Lindstrom E, et al Inhibition of cathepsin K reduces bone erosion, cartilage degradation and inflammation evoked by collagen-induced arthritis in mice. European journal of pharmacology. 2009;613(1–3):155–62. 10.1016/j.ejphar.2009.04.041 19358841

[42] Bhosale AM, Richardson JB. Articular cartilage: structure, injuries and review of management. British medical bulletin. 2008;87:77–95. 10.1093/bmb/ldn025 18676397

[43] Covington AD, Covington T. Tanning chemistry: the science of leather: Royal Society of Chemistry; 2009.

[44] Madhan B, Subramanian V, Rao JR, Nair BU, Ramasami T. Stabilization of collagen using plant polyphenol: role of catechin. Int J Biol Macromol. 2005;37(1–2):47–53. . 16183110 10.1016/j.ijbiomac.2005.08.005

[45] Tang HR, Covington AD, Hancock RA. Use of DSC To Detect the Heterogeneity of Hydrothermal Stability in the Polyphenol-Treated Collagen Matrix. Journal of agricultural and food chemistry. 2003;51(23):6652–6. 10.1021/jf034380u 14582955

[46] Schlebusch H, Kern D. Stabilization of collagen by polyphenols. Angiologica. 1972;9(3–6):248–52. . 4677552 10.1159/000157937

[47] Isenburg JC, Simionescu DT, Starcher BC, Vyavahare NR. Elastin stabilization for treatment of abdominal aortic aneurysms. Circulation. 2007;115(13):1729–37. 10.1161/Circulationaha.106.672873 WOS:000245402300009. 17372168

[48] Isenburg JC, Karamchandani NV, Simionescu DT, Vyavahare NR. Structural requirements for stabilization of vascular elastin by polyphenolic tannins. Biomaterials. 2006;27(19):3645–51. 10.1016/j.biomaterials.2006.02.016 WOS:000237125300015. 16527345

[49] Isenburg JC, Simionescu DT, Vyavahare NR. Elastin stabilization in cardiovascular implants: improved resistance to enzymatic degradation by treatment with tannic acid. Biomaterials. 2004;25(16):3293–302. 10.1016/j.biomaterials.2003.10.001 WOS:000220038500018. 14980424

[50] Isenburg JC, Simionescu DT, Vyavahare NR. Tannic acid treatment enhances biostability and reduces calcification of glutaraldehyde fixed aortic wall. Biomaterials. 2005;26(11):1237–45. 10.1016/j.biomaterials.2004.04.034 WOS:000225521700005. 15475053

[51] Tedder ME, Liao J, Weed B, Stabler C, Zhang H, Simionescu A, et al Stabilized Collagen Scaffolds for Heart Valve Tissue Engineering. Tissue Eng Pt A. 2009;15(6):1257–68. 10.1089/ten.tea.2008.0263 WOS:000266344600006. PMC2792094 18928400

[52] Chuang TH, Stabler C, Simionescu A, Simionescu DT. Polyphenol-Stabilized Tubular Elastin Scaffolds for Tissue Engineered Vascular Grafts. Tissue Eng Pt A. 2009;15(10):2837–51. 10.1089/ten.tea.2008.0394 WOS:000270553200008. PMC2792047 19254115

[53] Kasyanov V, Isenburg J, Draughn RA, Hazard S, Hodde J, Ozolanta I, et al Tannic acid mimicking dendrimers as small intestine submucosa stabilizing nanomordants. Biomaterials. 2006;27(5):745–51. 10.1016/j.biomaterials.2005.06.022 WOS:000233858000008. 16102811

[54] Liu Z, Zhou Q, Zhu JX, Xiao JH, Wan PX, Zhou CJ, et al Using genipin-crosslinked acellular porcine corneal stroma for cosmetic corneal lens implants. Biomaterials. 2012;33(30):7336–46. 10.1016/j.biomaterials.2012.06.080 WOS:000308524000003. 22795849

[55] Avila MY, Navia JL. Effect of genipin collagen crosslinking on porcine corneas. J Cataract Refr Surg. 2010;36(4):659–64. 10.1016/j.jcrs.2009.11.003 WOS:000276785200021. 20362860

[56] Fessel G, Cadby J, Wunderli S, van Weeren R, Snedeker JG. Dose- and time-dependent effects of genipin crosslinking on cell viability and tissue mechanics—Toward clinical application for tendon repair. Acta Biomater. 2014;10(5):1897–906. 10.1016/j.actbio.2013.12.048 WOS:000335095300012. 24384123

[57] Hiraishi N, Sono R, Sofiqul I, Yiu C, Nakamura H, Otsuki M, et al In vitro evaluation of plant-derived agents to preserve dentin collagen. Dent Mater. 2013;29(10):1048–54. 10.1016/j.dental.2013.07.015 WOS:000324237700009. 23942145

[58] Makris EA, Responte DJ, Paschos NK, Hu JC, Athanasiou KA. Developing functional musculoskeletal tissues through hypoxia and lysyl oxidase-induced collagen cross-linking. P Natl Acad Sci USA. 2014;111(45):E4832–E41. 10.1073/pnas.1414271111 WOS:000344526800007. 25349395 PMC4234579

[59] Satyam A, Subramanian GS, Raghunath M, Pandit A, Zeugolis DI. In vitro evaluation of Ficoll-enriched and genipin-stabilised collagen scaffolds. J Tissue Eng Regen M. 2014;8(3):233–41. 10.1002/Term.1522 WOS:000332973200008. 22552937

[60] Jackson JK, Zhao J, Wong W, Burt HM. The inhibition of collagenase induced degradation of collagen by the galloyl-containing polyphenols tannic acid, epigallocatechin gallate and epicatechin gallate. Journal of materials science. 2010;21(5):1435–43. 10.1007/s10856-010-4019-3 20162329

[61] Rasheed Z, Anbazhagan AN, Akhtar N, Ramamurthy S, Voss FR, Haqqi TM. Green tea polyphenol epigallocatechin-3-gallate inhibits advanced glycation end product-induced expression of tumor necrosis factor-alpha and matrix metalloproteinase-13 in human chondrocytes. Arthritis research & therapy. 2009;11(3):R71 . 19445683 10.1186/ar2700 PMC2714117

[62] Sung HW, Chang Y, Chiu CT, Chen CN, Liang HC. Mechanical properties of a porcine aortic valve fixed with a naturally occurring crosslinking agent. Biomaterials. 1999;20(19):1759–72. 10.1016/S0142-9612(99)00069-1 WOS:000082660400002. 10509186

[63] Larsen C, Ostergaard J, Larsen SW, Jensen H, Jacobsen S, Lindegaard C, et al Intra-Articular Depot Formulation Principles: Role in the Management of Postoperative Pain and Arthritic Disorders. J Pharm Sci-Us. 2008;97(11):4622–54. 10.1002/Jps.21346 WOS:000260607100003. 18306275

[64] Lay E, Samiric T, Handley CJ, Ilic MZ. Short- and long-term exposure of articular cartilage to curcumin or quercetin inhibits aggrecan loss. The Journal of nutritional biochemistry. 2012;23(2):106–12. 10.1016/j.jnutbio.2010.11.004 21419610

[65] Verzijl N, DeGroot J, Thorpe SR, Bank RA, Shaw JN, Lyons TJ, et al Effect of collagen turnover on the accumulation of advanced glycation end products. J Biol Chem. 2000;275(50):39027–31. . 10976109 10.1074/jbc.M006700200

