# Supplementary figures and images for: Intra-Articular Injections of Polyphenols Protect Articular Cartilage from Inflammation-Induced Degradation: Suggesting a Potential Role in Cartilage Therapeutics
Source: PLoS One. 2015 Jun 5;10(6):e0127165. doi: 10.1371/journal.pone.0127165 (PMC4457493; doi:10.1371/journal.pone.0127165)

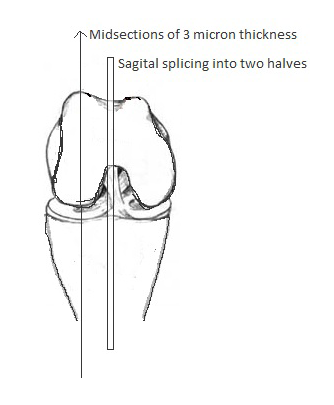

Supplement: S1 Fig — (TIF) [file pone.0127165.s001.tif]
